# Association Between Demodex Infestation and Ocular Surface Microbiota in Patients With Demodex Blepharitis

**DOI:** 10.3389/fmed.2020.592759

**Published:** 2020-11-04

**Authors:** Yan Yan, Qinke Yao, Yang Lu, Chunyi Shao, Hao Sun, Yimin Li, Yao Fu

**Affiliations:** ^1^Department of Ophthalmology, Shanghai Ninth People's Hospital Affiliated to Shanghai Jiaotong University School of Medicine, Shanghai, China; ^2^Shanghai Key Laboratory of Orbital Diseases and Ocular Oncology, Shanghai, China

**Keywords:** blepharitis, demodex, 16S, flora, micrbiota

## Abstract

**Purpose:** The aim of this study was to compare the ocular microbial communities in humans with and without demodex blepharitis in order to elucidate the relationship between ocular microorganisms and demodex infestation.

**Methods:** Bacterial 16S rRNA genes of conjunctival sac samples from 30 demodex blepharitis patients and 14 healthy controls were sequenced using a pyrosequencing method, and their bacterial community structures were compared by bioinformatics.

**Results:** Bacterial community clustering of conjunctival sac in the demodex blepharitis group were significantly distinct from the healthy control group, with significantly higher relative abundances of Firmicutes and *Corynebacterium* at the phyla level, as well as higher abundances of *Lactobacillus* and *Bifidobacterium* at the genus level. The relative abundance of *Staphylococcus epidermidis* (0.07–2.27%) was positively correlated with the demodex amount and modified OSDI. The major potential factors contribute to demodex blepharitis were Bacilli, Firmicutes, Cyanobacteria, *Lactobacillus* and Streptophyta.

**Conclusions:** Patients with demodex blepharitis have varying degrees of bacterial microbiota imbalance in the conjunctival sac. Demodex serving as vectors to transfer both skin and environmental flora might be the potential mechanism. In addition, the number and type of demodex affect the specific ocular surface bacteria, presenting as ocular discomfort and obvious signs of blepharitis.

## Introduction

Ocular demodex infestation is a very common but overlooked condition, causing various ocular surface diseases (1). The average incidence rate of mite infection is 13–70% worldwide (2–4). Moreover, demodex has been reported in the eyelashes of 18% of healthy individuals aged 21–35 years (5–7). Demodex blepharitis is a chronic inflammatory disease caused by demodex infestation, affecting the lid margin and ocular surface, which can lead to serious eye problems (1). The typical symptoms include eye itching, burning, dryness, irritation, watering, blurry vision, or the sensation of heavy eyelids (8).

However, the role of the demodex mite as an etiologic factor of chronic blepharitis has remained controversial for many years (9, 10). *Demodex folliculorum*, and *Demodex brevis* are the two primary types of demodex identified in humans that are associated with blepharitis, meibomian gland dysfunction and dry eye diseases (1, 11, 12). At present, demodex are mainly thought to be mechanical carriers of pathogenic bacteria, including *Staphylococcus* and *Streptococcus*. The therapeutic strategies against demodex are based on reducing or eradicating the parasites, and include topical application of topical tea tree oil (TTO) and metronidazole ointment. However, significant alterations of tear film and tear cytokine levels have been observed in patients with demodex blepharitis (13, 14). Even if the demodex die, the pathogenic bacteria they carry can continue to cause inflammatory reactions, so anti-inflammatory treatment is equally important as mite reduction treatment. The efficacy of different treatments for Demodex blepharitis has been a research hotspot for several years (15). There is a lack of definite proof on whether there are changes in the ocular surface microbiota in patients with demodex, and there are few studies on the effects of mites on ocular surface flora.

Exploring the effect of mites on ocular surface bacteria can help to clarify the pathological mechanism as well as improve the treatment strategies of demodex blepharitis. Higher abundances of Streptophyta, *Corynebacterium*, and *Enhydrobacter* were found in tear samples and eyelashes of seven blepharitis patients with an average age of 67 years. However, the correlation between demodex and ocular microbial community remains unclear (16). With the traditional cultivation, *Propionibacterium acnes* colonies increased significantly in eyelashes of demodex blepharitis patients (*D. folliculorum*), which laid a foundation for the study of demodex (17). More recently, Ocular bacterial communities have been studied with 16S rRNA gene sequencing in healthy subjects (18) and in patients with ocular diseases. Thus, defining the characteristics of bacterial community on the ocular surface of patients with demodex blepharitis based on 16S rDNA sequencing technology may promote further investigations on the role of ocular demodex, and provide valuable information for the prevention as well as treatment of human demodex blepharitis.

## Materials and Methods

### Study Population

A total of 44 participants were recruited between July 2019 and May 2020, including 30 patients with demodex blepharitis (eight males and 22 females, aged 41.07 ± 16.03 years) and 14 age and sex-matched healthy controls (three males and 11 females, aged 41.14 ± 15.81 years). Patients who had used topical or systemic antibiotics within 1 month, with a history of ophthalmic surgeries, other ocular diseases that required priority treatment, incomplete medical patient records, age >65 years, and patients with severe systemic diseases were excluded from this study. The study was approved by the Ethics Committee of Shanghai Ninth People's Hospital and complied with the tenets of the Declaration of Helsinki for clinical research (IRB: SH9H-2019-T307-2). Written informed consent was obtained from all participants after explaining the purpose and possible consequences of the study.

### Diagnosis of Demodex Blepharitis

**Figure 1:**
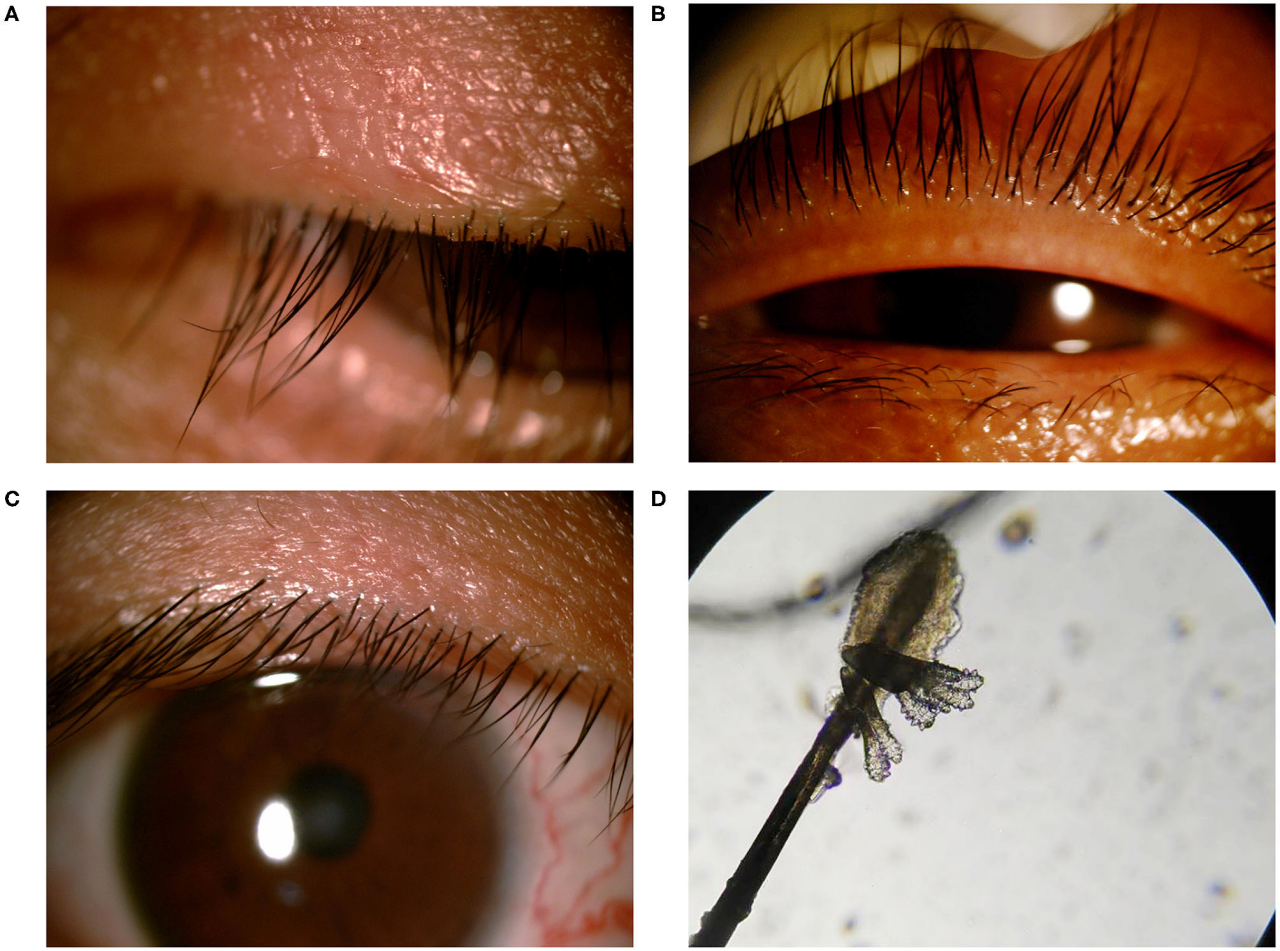
Standard external photographs of eyelids of patients with blepharitis and demodex. Clinical features of lashes with severe cylindrical dandruff **(A)**. Image of meibomian gland occlusion in patient with ocular demodecosis **(B)**. Image of marginal hyperemia in patient with non-Demodex blepharitis **(C)**. *Demodex folliculorum* (magnification 40x) **(D)**.

The diagnosis of demodex blepharitis was made based on symptoms, clinical signs, and ophthalmologic examinations. All patients underwent complete ophthalmologic examinations. Blepharitis was diagnosed by evidence of lid margin or tarsal conjunctival erythema, bulbar conjunctival hyperemia, telangiectasias, thickening, or irregularity of the eyelid margins, or meibomian gland orifice inclusions (Figures 1A–C). Ocular discomfort was assessed by modified ocular surface disease index (OSDI) (19). The severity of blepharitis was assessed by Efron grading scales (20). The patients with suspected demodex infection as evidenced by cylindrical dandruff were confirmed by microscopic examination. The modified eyelashes examination was conducted as previously reported (21, 22). Briefly, two lashes were removed from each lid by fine forceps under a slit-lamp biomicroscope and placed separately on each end of a glass slide, with a total of eight lashes on four slides. One drop of saline was applied by a pipette to the edge of the coverslip for lashes without retained CD, and one drop of fluorescein solution was added for those with retained CD to allow embedded Demodex to migrate out. If a compacted CD was preserved, 20 μL of 100% alcohol was pipetted into the edge of the coverslip, and the counting time was prolonged up to 20 min to allow the embedded Demodex to migrate from the CD. Under the microscope, a positive result involved the presence of at least three Demodex bodies, including adult, larva, protonymph, or nymph stage of *D. folliculorum* or *D. brevis* (Figure 1D). In parallel, the number and species of Demodex were counted. We defined Group A as demodex blepharitis patients and Group B as healthy controls.

### Sample Collection

Subjects were sampled with the selected eye examined for more demodex. After topical anesthesia with 0.4% oxybuprocaine hydrochloride eye drops (Santen, Osaka, Japan), a sterile dry cotton swab was used to wipe both the upper and lower conjunctival sacs. The procedure was repeated twice. Two blank sterile swabs were also collected. Each swab was immediately placed into a sterile tube and stored in an ultra-low temperature freezer at −80°C before DNA extraction.

### DNA Extraction and Sequencing

DNA was extracted from all samples using the PowerMax soil DNA isolation kit (MoBio Laboratories, Carlsbad, CA, USA), according to the manufacturer's instructions. Blank samples also underwent a complete extraction procedure to exclude false-positive results. The V3-V4 region of 16s rRNA gene was amplified by PCR with Phusion high fidelity DNA polymerase (NEB, UK) and the primer sets 338F (5′-ACTCCTACGGGAGGCAGCA-3′), 806R (5′-GGACTACHVGGGTWTCTAAT-3′), as described in Supplementary Material. Lastly, all the samples were sequenced on an Illumina MiSeq platform (Illumina, Inc.) and 250-bp paired-end reads were generated. The results were saved in FASTQ (.fq) format, which contained sequence information of the reads and the corresponding quality information.

### Data Processing and Analysis

The raw data were first spliced (FLASH[c], version 1.2.11), and quality trimming was applied to the spliced sequence (Trimmomatic, version 0.33) to remove the chimeras (UCHIME, version 8.1), in order to obtain high-quality sequence tags, as previously reported (23–25). Sequences with a similarity >97% were classified as the same operational taxonomic units (OTUs) by USEARCH, version 10.0, and 0.005% of all sequences was used as the threshold to filter OTUs (26). Taxonomy was assigned using the Greengene as the reference database. The alpha and beta diversities of all groups were also obtained by calculation and analysis. Linear discriminant analysis effect size (LEfSe) was introduced to identify bacterial biomarkers of the healthy control group and the demodex blepharitis group.

### Statistical Analyses

The data were statistically analyzed using SPSS 15.0 software (Chicago, IL, USA). Student's *t*-test and Mann-Whitney U-test were used to compare the differences in age, gender, ethnicity, and clinical examination results between the patients with MGD and the controls. Mann-Whitney U-test was performed for analyses of the adversity indices and the relative abundances of dominant phyla and genera between the groups. Spearman's correlation analysis was used to measure the correlation between meibo scores and relative abundances of *Staphylococcus* in patients with MGD. A *p*-value < 0.05 was considered statistically significant.

## Result

### Demographics and Clinical Features of the Study Participants

**Figure 2:**
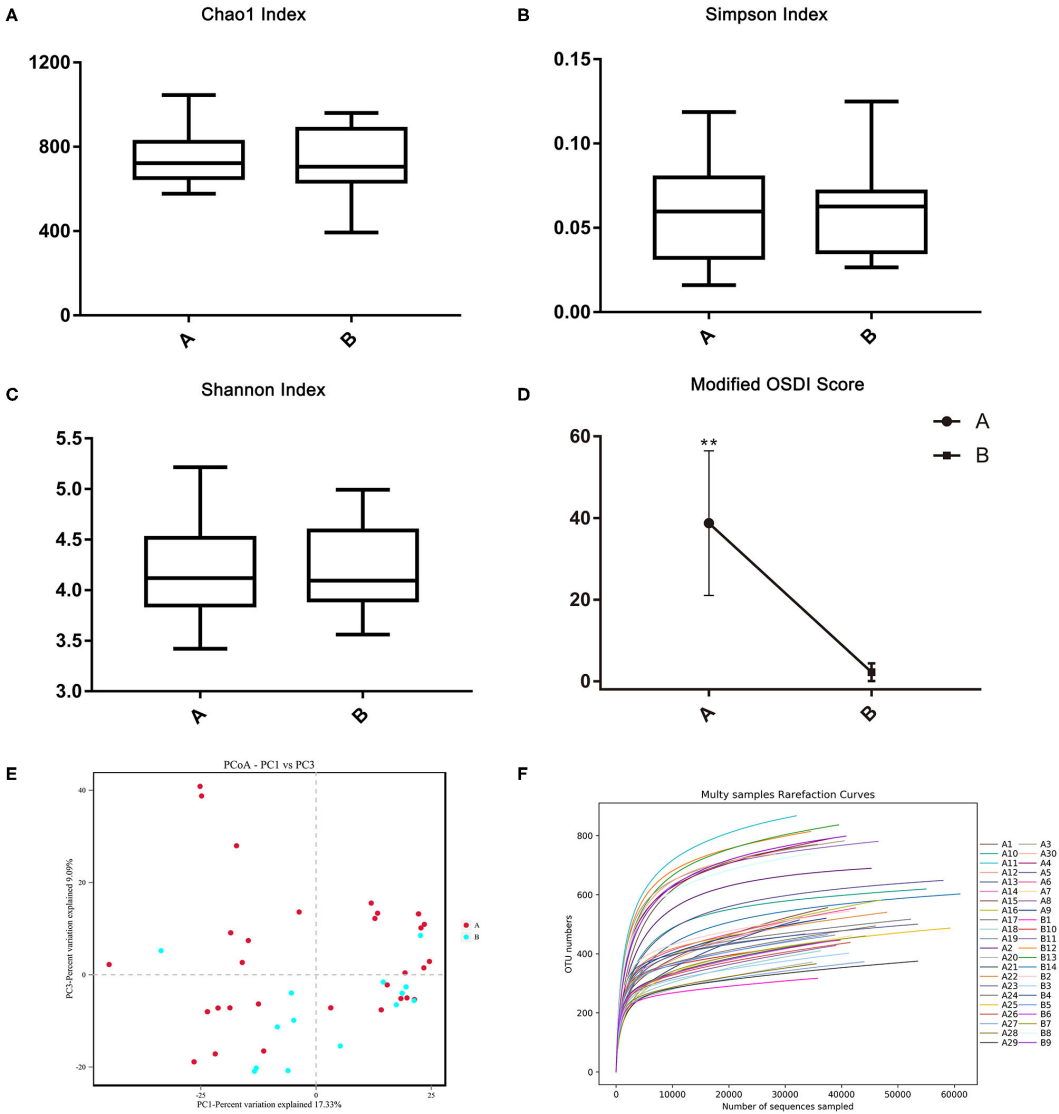
The alpha and beta diversities between the demodex blepharitis patients and healthy controls. The Chao1 **(A)**, Simpson **(B)**, and Shannon index **(C)** representing the alpha diversity. The principal coordinate analysis was constructed using the Bray-Curtis method **(E)**. Modified OSDI scores of patients with and without demodex blepharitis. The modified OSDI scores of the demodex blepharitis patients were significantly higher than those of the healthy controls (^**^p < 0.05) **(D)**. The multi-samples refraction curves. Steep slopes of the rarefaction curves of individual samples **(F)**.

A total of 30 patients (22 females and 8 males, aged 41.07 ± 16.03 years) and 14 normal individuals (11 females and three males, aged 41.14 ± 15.81 years), matched by age and gender, were consecutively recruited in this study. The average score of modified OSDI in the demodex blepharitis patients was significantly higher than that in the healthy controls (38.72 ± 17.03, 9.74 ± 5.23, *p* < 0.05, Independent-sample *t*-test) (Figure 2D). The results of Efron Grading Scale and average number of demodex mites on 8 lashes are shown in Table 1.

**Table 1:** Basic information of patients with demodex blepharitis and healthy controls.

		**Demodex blepharitis patients**	**Healthy controls**
Gender	Female	22	11
	Male	8	3
Age		41.07 ± 16.03	41.14 ± 15.81
Modified OSDI		38.72 ± 17.03^*^	9.74 ± 5.23
Efron grading scale	Grade 2	20	/
	Grade 3	10	/
Demodex	*D. folliculorum*	34	/
	*D. brevis*	8	/

* *p < 0.05, Independent-sample t-test. OSDI, ocular surface disease index*.

### Bacterial Diversity in Samples

From the pyrosequencing of 44 PCR amplicons, a total of 3,412,282 sequencing reads was generated. After the removal of low-quality or non-bacterial 16S rRNA sequencing reads, 3,190,237 high-quality reads (93.49% of the total reads) for each sample were used for further analysis.

There was no significant difference in the Chao1 index, Simpson index, and Shannon index (Figures 2A–C) between the two groups (*p* > 0.05, Independent-Samples *t*-test). Steep slopes of the rarefaction curves of individual samples (Figure 2F) suggested that the majority of the species diversities were discovered.

The Chao1 index, Simpson index, and Shannon index were not influenced by gender (Independent-Samples *t*-test) and age (ANOVA), but were influenced by species of demodex (multiple linear regression analysis, *p* < 0.05). Moreover, the Chao1 index was influenced by the number of demodex (multiple linear regression analysis, *p* < 0.05). Spearman's rank-order correlation was used to analyze each variable, increased species of demodex was significantly correlated with Simpson index (*r* = 0.39, *p* = 0.033) and Shannon index (*r* = −0.443, *p* = 0.014).

Through the phylogenetic and distance matrix, principal coordinates analysis (PCoA) rearranged the samples in low-dimensional space according to their resemblance indices. Usually 2-3 eigenvalues, which accounted for more than 50% differentiations of data were selected to establish the coordinates for visualization of the similarities among the samples. The analysis gathered samples with high community structural similarity, while samples with large community structural differences were far apart. Most of the samples from the demodex blepharitis group were clustered far apart from the normal healthy subjects (Figure 2E). This indicated significant difference in the bacterial community composition between the demodex blepharitis group and the healthy group.

### Bacterial Taxonomy and LEfSe Analysis

The demodex blepharitis and healthy groups were classified into six significant bacterial phyla: Firmicutes (41.71; 29.30%), Proteobacteria (20.51; 26.96%), Actinobacteria (20.66; 23.87%), Bacteroidetes (7.95; 11.04%), Cyanobacteria (4.80; 3.81%), and Chloroflexi (0.92; 1.41%) (Figure 4). The relative abundances of Firmicutes (*p* < 0.01, Mann-Whitney U-test), and Cyanobacteria (*p* < 0.05, Mann-Whitney U-test) in the healthy controls were significantly lower than the demodex blepharitis group.

**Figure 3:**
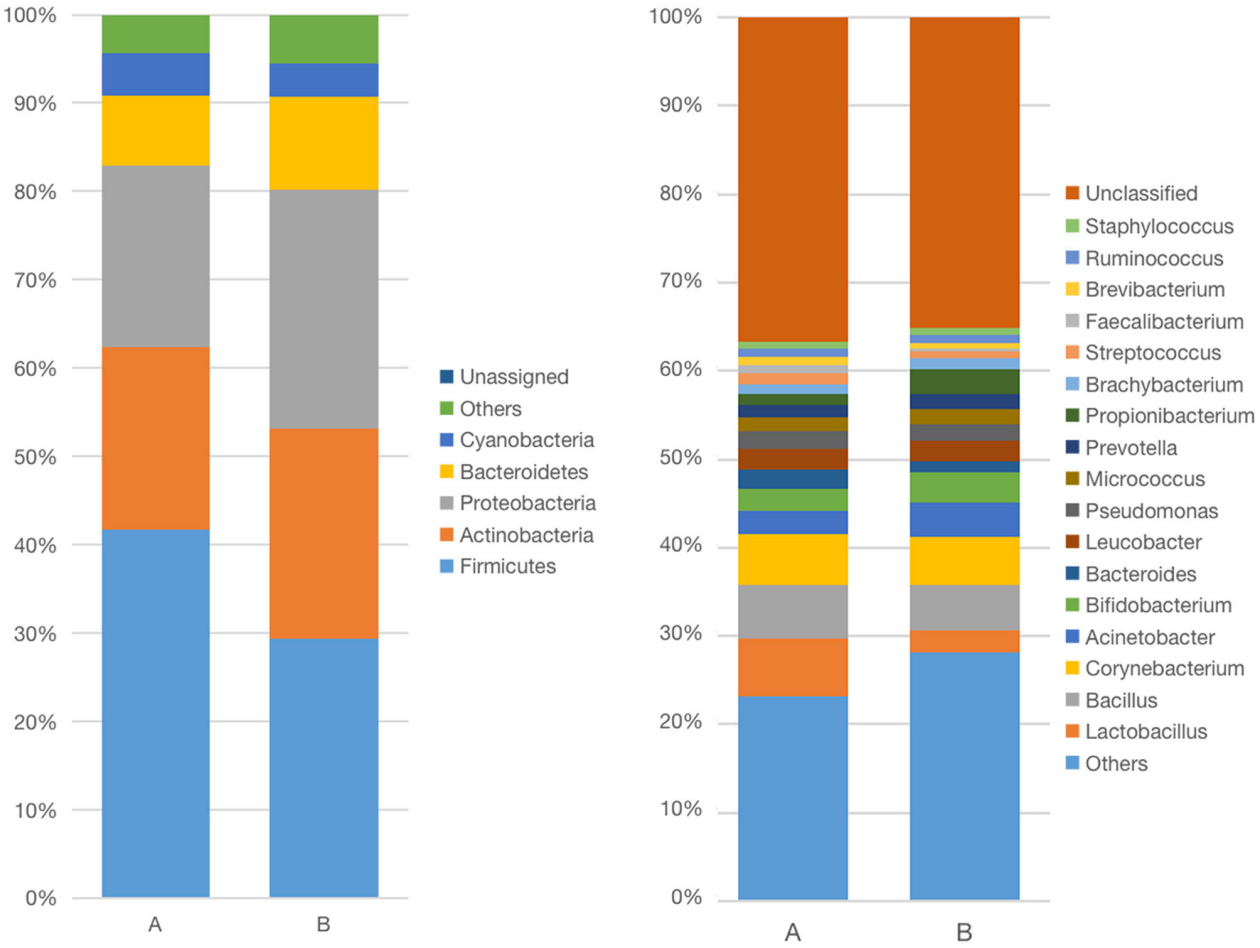
Differences in relative mean abundances of phylotypes in ocular microbiota between the demodex blepharitis patients and healthy controls. Each phylotype (1% of average relative abundance in groups) is indicated by a different color at the genus level (A) and phylum level (B).

Moreover, at the genus level, both groups were categorized into 16 bacterial genera. The most predominant bacteria were *Lactobacillus* (6.43; 2.48%), *Bacillus* (6.13; 5.04%), *Corynebacterium* (5.93; 5.47%), *Acinetobacter* (2.69; 3.90%), *Bacteroides* (2.36; 1.21%), *Streptococcus* (1.13; 0.85%), *Bifidobacterium* (2.36; 3.44%), *Pseudomonas* (1.99; 1.88%), *Propionibacterium* (1.26; 2.83%), and *Micrococcus* (1.59; 1.70%) (Figure 3). Furthermore, *Bacillus* belong to phyla of Firmicutes and was abundant in genera. Compared with the healthy group, the demodex blepharitis group had a significantly higher abundance of *Lactobacillus* (*p* < 0.01, Mann-Whitney U-test) and *Bifidobacterium* (*p* = 0.05, Mann-Whitney U-test). There was no significant difference in the abundance of *Streptococcus* (*p* > 0.05, Mann-Whitney U-test) or *Propionibacterium* (*p* > 0.05, Mann-Whitney U-test).

**Figure 4:**
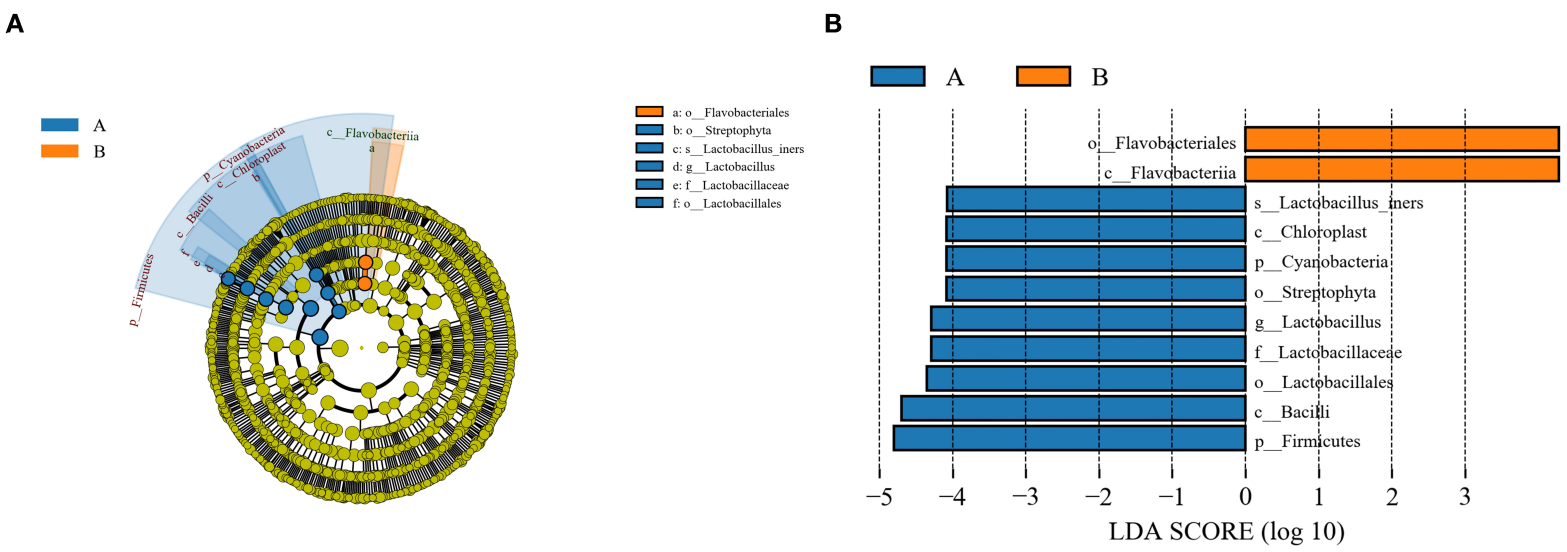
LEfSe analysis of the normal control group and the MGD group. A cladogram of the conjunctival sac bacterial taxa in the patients with demodex blepharitis (green) and the control group (orange) showed the levels from domain to species and from outside to inside **(A)**. Taxa listed according to their linear discriminant analysis (LDA) values determined from comparisons between the blepharitis patients and healthy subjects in the DE group as computed by the LEfSe algorithm **(B)**.

As a high-dimensional class comparison to determine the operational taxon that is most likely to explain the differences between the two groups, LEfSe was employed to identify the most potentially pathogenic bacterial biomarkers by combining statistical significance with the consistency and effect correlation of coding organisms (Figure 4). The biomarkers were Lactobacillaceae, Streptophyta (family), Bacilli (class), Firmicutes, Cyanobacteria (phyla), and *Lactobacillus* (genera) in the demodex blepharitis group, and Flavobacteriia (class), Flavobacteriales (order) in the healthy control group (LDA > 4).

### Association With Clinical Parameters

At the species level, the relative abundance of *Staphylococcus epidermidis* (0.07–2.27%) was significantly related to the demodex amount (*R* = 0.55, *p* = 0.002), modified OSDI (*R* = 0.448, *p* = 0.013, Pearson's correlation), Efron Grading Scale (*R* = 0.434, *p* = 0.017, Pearson's correlation) and age (*R* = 0.404, *p* = 0.027, Pearson's correlation). *Pseudomonas viridiflava* and Weissella paramesenteroides were significantly affected by demodex species (*p* = 0.001, Pearson's correlation). Moreover, demodex amounts were significantly related to modified OSDI (*R* = 0.693, *p* < 0.001, Pearson's correlation), Efron Grading Scale (*R* = 0.480, *p* = 0.007, Pearson's correlation), and age (*R* = 0.526, *p* = 0.003, Pearson's correlation). The species of demodex was not affected by other clinical parameters.

## Discussion

Chronic infestation of demodex in the eyelids may result in inflammation of the ocular surface and secondary bacterial infection (19). Thorough understanding of the characteristics of ocular microbial community associated with demodex is essential for the pathogenesis, prevention, and treatment of blepharitis. Zhu et al. showed that the total colony counts from blepharitis patients' eyelashes were significantly higher than that of the healthy controls (17). Because bacterial culture methods have limitations in bacterial identification, we compared the conjunctival sac bacterial microbiota between subjects with and without demodex blepharitis using 16S rRNA gene sequencing in this study.

The most prevalent microbes on the ocular surface were similar to the skin flora. Previous studies reported that mixed skin microbial flora, including *P. acnes*, were found in blepharitis patients and healthy controls, but *Staphylococcus* and other skin microbial flora were not found in healthy controls (17). Lee et al. reported that the relative abundances of Streptophyta, *Corynebacterium*, and *Enhydrobacter* were higher on the ocular surfaces of subjects with blepharitis (*n* = 7) than in healthy subjects (16). These previous reports suggested that human blepharitis might be induced by infestations of mixed skin microbial flora. This study demonstrated that Firmicutes, Proteobacteria, Actinobacteria, Bacteroidetes, and Cyanobacteria were the main flora in patients with and without demodex blepharitis. Consistently, the presence of *Bacillus, Staphylococcus, Streptococcus, Propionibacterium*, and *Corynebacterium* in cultures from demodex blepharitis patients has been previously reported (17). We also detected members of other skin taxa in patients with demodex infestation, such as *Lactobacillus, Bacteroides, Bifidobacterium, Micrococcus*, and *Acinetobacter* at a relative abundance of 1% in more than half of the samples.

Moreover the relative proportions of Cyanobacteria, whose source may be dust, pollen or plant material, were clearly higher in patients with blepharitis than in healthy controls. As reported in previous studies, the hypothesis was that blepharitis might be induced by pollens or pollution (16). However in this study, Cyanobacteria was identified as the most potentially pathogenic bacterial biomarker by LEfSe. Given that demodex infestation was more common in patients who had pets or lived in poor sanitary conditions, these people inevitably rub their eyes to relief ocular discomfort. The ocular surface flora of patients with demodex blepharitis could be significantly affected by environmental factors, such as the bacteria from soil and dust. Representatives of these genera are considered to be opportunistic pathogens in conjunctivitis and keratitis. Enrichment of skin and environmental bacteria caused by demodex infestation suggests that demodex could function as a vector to transfer both skin and environmental bacteria to the ocular surface.

Demodex mites are the most common permanent ectoparasites in human skin (27). The epidemiological and clinical correlation between demodex infestation of the eyelids and blepharitis has been investigated for decades. Some prior studies have reported that demodex-related *Bacillus* might contribute to the occurrence of blepharitis (28–31). In this study, *Bacillus* was also detected as a potentially pathogenic bacterial biomarker of demodex infestation in eyes. In addition, *S. epidermidis* had a positive correlation with the amount of demodex and the severity of ocular symptoms and signs, which further confirmed the relationship between demodex and blepharitis. Clinically, we have found that patients with *D. brevis* often required a longer course of treatment. In this study, *Pseudomonas viridiflava*, which is mostly found in soil, was significantly related to *D. brevis*. Therefore, patients with *D. brevis* had higher abundance of environmental pathogens, and need more thorough lid cleaning, or additional anti-inflammatory treatment. These results provided additional evidence to support the hypothesis that a high prevalence of demodex mite infestation is accompanied by higher abundance of certain bacteria on the ocular surface. Also, the results further confirmed that demodex infection was strongly linked to imbalances in the species of bacteria.

As the traditional treatment of demodex blepharitis emphasizes solely on killing mites, this study highlighted the importance of additional anti-bacterial treatment on the ocular surface along with lid hygiene, which is more appropriate and comprehensive. Meanwhile, doctors and nurses should pay more attention to health education to improve patients' hygiene habits, such as not rubbing their eyes with hands.

Further studies are needed to identify the opportunistic pathogens associated with demodex infections in eyes, as well as to compare the microbial communities in patients with blepharitis with and without demodex infestation. And also the Next-generation sequencing technology has the intrinsic bias of excess background noise, which may potentially undermine the conclusions and limits clinical applicability. Moreover, the anesthetic may have diluted or washed away bacteria from the ocular surface, which should be avoided by better sample collection method. Despite these limitations, this study provided novel insights into possible mechanisms by which demodex carry both skin and environmental bacteria causing inflammation. Moreover, the number and type of demodex affect the specific ocular surface bacteria, leading to ocular discomfort and worsening of blepharitis signs, which provides a reliable basis for physicians to optimize treatment plans.

## Data Availability Statement

The datasets generated for this study can be found in NCBI Sequence Read Archive, PRJNA657256.

## Ethics Statement

The studies involving human participants were reviewed and approved by the Ethics Committee of Shanghai Ninth People's Hospital (IRB: SH9H-2019-T307-2). The patients/participants provided their written informed consent to participate in this study.

## Author Contributions

YY, YF, QY, and YLi conceived and designed the experiments. YY, YLu, CS, and YLi performed the experiments. YY and YLi analyzed the data. YY, QY, and YF contributed the reagents, materials, and analysis tools. YY, YLi, HS, QY, and YF wrote the manuscript. All authors contributed to the article and approved the submitted version.

## Conflict of Interest

The authors declare that the research was conducted in the absence of any commercial or financial relationships that could be construed as a potential conflict of interest.

## Supplementary Material

Supplementary Material DNA sequencing methods.
