# Supplementary material for: Association Between Demodex Infestation and Ocular Surface Microbiota in Patients With Demodex Blepharitis
Source: Front Med (Lausanne). 2020 Nov 4;7:592759. doi: 10.3389/fmed.2020.592759 (PMC7672197; doi:10.3389/fmed.2020.592759)
Supplement: Supplementary Material — DNA sequencing methods. [file Data_Sheet_1.docx]

Supplementary Material

**PCR amplification of clinical samples**The V3-V4 variable regions of bacterial 16S rRNA of the 89 samples were amplified with the primers 338F (5'-ACTCCTACGGGAGGCAGCA-3') and 806R (5'-GGACTACHVGGGTWTCTAAT-3'). The 5’ ends of the primers were tagged with specific barcodes and universal primers for sequencing. All reactions were carried out in 50 μl mixtures [template DNA 40 ng, Vn F (10 μM) 1.5 μl, Vn R (10 μM) 1.5 μl, Q5 High-Fidelity DNA polymerase 0.2 μl, High GC enhancer 10 μl, Buffer 10 μl, dNTP 1 μl and ddH2O] for amplification of bacterial 16S fragments via degeneration, denaturation, annealing, extension, and final extension. The detailed procedure of PCR reactions is listed in the Table.

| **Cycle** | **temperature** | **time** |
| --- | --- | --- |
| 30 | 98°C | 2 min |
|  | 98°C | 30s |
|  | 50°C | 30s |
|  | 72°C | 1min |
| 1 | 72°C | 5min |
|  | 4°C | ∞ |

The PCR products were examined using agarose gel electrophoresis (AGE). A 2% gel was used to identify the PCR products. MinElute® PCR purification kit was used to purify the products and quantified with a NanoDrop ND2000 spectrophotometer (Thermo Scientific, Wilmington, DE, USA).

Finally, the quantity of DNA was assessed using 1.8% AGE. Sequencing libraries were quantified using Qubit and then pooled to obtain a sufficient concentration.

Data processing

For paired-end read splicing, FLASH v1.2.11 software was used. According to the minimum overlap length of 10 bp and the permitted maximum mismatch ratio of 0.2 (default) in the overlap, the reads of each sample were spliced to obtain the spliced sequence, i.e., the raw tags. The operating principle of Trimmomatic is as follows. Trimmomatic is a flexible trimmer for Illumina sequence data. It can be used for quality trimming of double-ended sequencing or single-ended sequencing data in the FASTQ format, with the base quality of phred 33 or phred 64 (depending on the Illumina sequencing machine). The parameters were set as follows: The length of the window was 50 bp. If the mean quality value within the window was <20, the back bases were truncated from the window, and the tags whose quality control was <75% of the length of the tags were filtered to obtain high-quality tag data, i.e., clean tags. The principle of UCHIME is shown in the figure below. In the first step, the query sequence is split into non-overlapping chunks, and then the chunks are compared with the database. The second step is to select the best match for each chunk in the database and ultimately select the two best parent sequences. In the third step, the query sequence to be detected is compared with those two parent sequences. If there is a sequence in each of the two parent sequences whose similarity with the query sequence is >80%, the query is determined to be a chimera. All samples are publicly available at NCBI-SRA:PRJNA657256.
